# Changes in and relationships between human milk oligosaccharides and microRNAs in milk-derived extracellular vesicles during the first 4 months of lactation

**DOI:** 10.3389/fnut.2025.1694093

**Published:** 2025-12-22

**Authors:** Mai Morozumi, Hirohisa Izumi, Muneya Tsuda, Fuka Tabata, Hirohiko Nakamura, Kazuhiro Miyaji

**Affiliations:** Health Care & Nutritional Science Institute, Morinaga Milk Industry Co., Ltd., Zama, Kanagawa, Japan

**Keywords:** human milk oligosaccharides, extracellular vesicles, microRNA, breast milk, Japanese

## Abstract

**Background:**

Human milk oligosaccharides (HMOs) exert beneficial effects on the gut microbiota, enhance resistance to infections, support immune development, and contribute to brain/cognitive development. Milk-derived extracellular vesicles (MEVs) contain a high abundance of immunity- and development-related microRNAs (miRNAs). These components are abundant in breast milk. In the case of HMOs, the composition varies due to factors such as lactation stages, geographic location, ethnicity, genetics, and the environment. The composition of HMOs is significantly influenced by the genetic status of two key genes: FUT2 (Secretor gene) and FUT3 (Lewis gene). In this study, we broadly categorized them as secretors or non-secretors.

**Methods:**

We investigated the changes in the concentrations of HMOs and MEVs during 4 months of lactation in Japanese women and explored the relationship between HMOs and miRNAs present in MEVs.

**Results:**

The concentrations of most HMOs significantly decreased over time. The number of MEVs did not change significantly over the study period. Interestingly, 3′-sialyllactose and lacto-N-fucopentaose III were inversely correlated with many of the top 20 most abundant miRNAs. Moreover, miRNAs in MEVs, which are associated with immunity and development, were more abundant in secretors than in non-secretors during early lactation. Several HMOs were detected in MEVs.

**Conclusion:**

This study enabled a detailed characterization of changes in HMOs and MEVs in the breast milk of Japanese women throughout the course of the first 4 months of lactation. A potential association between the concentrations of HMOs and miRNAs was also observed, suggesting that these components might influence each other. These findings are significant for promoting healthy infant development and growth, as well as for improving infant formula composition.

## Introduction

Breast milk is an optimal nutrient source for infants and plays a significant role in their development by contributing to the prevention of infections and malocclusion, enhancing cognitive development, and reducing the risk of obesity and diabetes (1). It is highly complex, and the functions of its bioactive components are not fully understood. Breast milk and infant formula differ in their effects on immunocompetence (2, 3). Infants who exclusively breastfed for 4 to 6 months had a fourfold increase in the risk of pneumonia compared with infants who exclusively breastfed for more than 6 months (4). Moreover, the gut microbiota, which is closely related to metabolic and immune functions (5, 6), differs between breastfed and formula-fed infants (7). However, in some cases, breastfeeding is inadequate because of medical factors, adoption, and/or the choice of the mother not to breastfeed. The majority of formula is produced from cow’s milk, and reproducing the composition of breast milk is problematic. Information on the changes and functions of the beneficial bioactive components in breast milk over time would enable bridging of the gap between breast- and formula-fed infants.

Human milk oligosaccharides (HMOs) are complex glycans that are highly abundant in breast milk, comprising 4%–11% of its solid components, following lactose and fat (8). HMOs exert beneficial effects on the gut microbiota, resistance to infections, immune development, and brain and cognitive development (9). HMOs comprise glucose, galactose, N-acetylglucosamine, fucose, and sialic acid in various proportions, resulting in diverse pools of complex oligosaccharides. Over 150 structurally unique HMOs have been identified, and the composition of HMOs differs according to geographic, ethnic, genetic, and environmental factors (10). In spite of the identification of these factors that influence the composition of HMOs, their biosynthesis and the determinants of their concentration have not yet been fully elucidated. Approximately 75% of the breastfeeding population are classified as secretors; the other 25% are classified as non-secretors (11). Secretors have an active secretor gene that encodes the enzyme α1–2-fucosyltransferase (FUT2). As a result, they have high concentrations of 2′-fucosyllactose (2′FL), lacto-N-fucopentaose I (LNFPI), and other α1–2-fucosylated HMOs, whereas non-secretors have high concentrations of fucosylated HMOs, with the exception of α1–2-fucosylated HMOs such as lacto-N-fucopentaose II (LNFPII) and lacto-N-fucopentaose III (LNFPIII). As a consequence, the breast milk of secretors and that of non-secretors have different HMO compositions. There are differences in weight and microbiota composition between breastfed infants of secretors and those of non-secretors (9). Longitudinal changes in HMOs have been investigated in Italy, Brazil, Singapore, Germany, and Spain (12–16), but few studies have tracked the changes in HMOs over time in Japan (17). HMOs have been historically difficult to measure and synthesize (8, 18), resulting in few efforts to determine functions or concentrations of various HMOs in Japanese breast milk. Given the uncertainty over the HMO concentrations in Japanese and the uncertainty of their functions, investigating the changes in HMO levels throughout lactation in Japanese women would provide insight into the geographical variations in HMO profiles and facilitate the development of beneficial infant formulae.

Breast milk contains various bioactive components, including extracellular vesicles (EVs). EVs are nanovesicles (20–200 nm) with an endosome-derived limiting membrane secreted by diverse cell types and are abundant in breast milk. EVs transport proteins, lipids, DNA, and various types of RNAs [such as mRNAs, long non-coding RNAs, and microRNAs (miRNAs)]. Milk-derived EVs (MEVs) contain a high abundance of immunity- and development-related miRNAs (19). MEVs, which transmit signals from the mother to offspring, are related to intestinal diseases and the immune system in infants (20). MEVs can prevent necrotizing enterocolitis by reducing inflammation and injury to the intestinal epithelium and restoring intestinal tight junctions (21). MEVs inhibit HIV-1 infection of dendritic cells (22). These findings suggest that maternal MEVs are important for offspring health. The separation of MEVs from milk is more difficult than from other body fluids because of the presence of components such as casein. As a result, the changes in the concentrations and/or functions of MEVs during lactation are unclear. We previously reported a simple and optimal method for isolating EVs from cow milk (23). In this study, we used the method to collect EVs from human breast milk (23). To evaluate their biological functions, it is important to understand the changes in the concentrations of MEVs and miRNAs in individuals over the course of lactation. However, few studies have focused on this matter; even fewer studies have involved Japanese subjects.

Although no conclusion has been reached, it has been reported that immune capability may differ according to the secretor type. Factors contributing to this difference in immune capability are discussed. We considered that there might be immune factors related to secretor type beyond HMOs. Recently, some HMOs have been detected in MEV (24), and patterns of miRNAs in MEV levels summarized using principal components were associated with HMO summary measures and concentrations (25). These data suggest an association between MEVs and HMOs; therefore, our analysis examined the association between HMOs and miRNAs.

We investigated the changes in HMOs and EVs in breast milk over time in Japanese women. Examining fluctuations at detailed time points during the lactation period enables us to observe subtle changes that are difficult to detect in large-scale studies. We hypothesized that secretor type could influence the composition of miRNAs in MEVs. Understanding this relationship would provide insight into which HMOs and MEVs are important during critical periods of infant development. This is the first investigation to examine the relationships between miRNAs in MEVs and secretor types, as well as the temporal changes in HMOs and MEVs in Japanese women. The data indicate the importance of the relationships between HMOs and miRNAs in MEVs and will facilitate improvements of infant formula with respect to HMOs and MEV content.

## Materials and methods

### Subjects

This study was conducted in accordance with the principles of the Declaration of Helsinki and was approved by the Institutional Review Board of the Japan Conference of Clinical Research (Protocol No. BONYU-01). Participants were primarily employees of Morinaga Milk Industry, as well as their family members and friends. The study was conducted among healthy pregnant and lactating women. Breast milk was supplied by 16 volunteers, and written informed consent was obtained from them before enrollment; *n* = 12 for colostrum, *n* = 12 for transition, *n* = 14 for 1 month, *n* = 14 for 2 months, *n* = 13 for 3 months, and *n* = 11 for 4 months.

### Milk collection

Participants were encouraged to pump the entire contents of a full breast, which included fore-, mid-, and hind-milk. Samples were generally collected at home and stored at −20 °C. The duration of home storage ranged from a few days to up to 9 months. Study personnel subsequently retrieved the samples, which were then stored at −80 °C until analysis.

### Analysis of human milk composition

The major nutrients in breast milk were analyzed using the Miris Human Milk Analyzer (Miris, Uppsala, Sweden), which enables simultaneous measurement of the concentrations of true protein, fat, carbohydrates, and total solids. The macronutrient content of milk was used to calculate digestible energy. Before analysis, each sample was warmed to 40 °C and homogenized.

### Analysis of HMOs

HMOs were analyzed by liquid chromatography–tandem mass spectrometry (LC–MS; Q Exactive Orbitrap; Thermo Fisher Scientific, Waltham, MA). Details of the measurements are described below. Breast milk samples were diluted at a 1:16 ratio with stachyose solution. Breast milk was spiked with stachyose (Tokyo Chemical Industry, Tokyo, Japan) at the beginning of sample preparation to correct for sample loss during sample processing. The samples were centrifuged at 4,500 × *g* for 10 min at 4 °C to collect the middle layer, and 1.5 volumes of acetonitrile were added. The samples were then centrifuged at 21,500 × *g* for 15 min at 4 °C, and the supernatants were collected (26). The supernatants were passed through a 0.22-μm filter, and the filtrate was subjected to LC–MS. HMOs were analyzed by LC–MS on an Acquity Glycoprotein BEH Amide column (300Å, 1.7 μm, 2.1 × 150 mm; Waters, Milford, MA). Mobile phase A was 10 mM ammonium formate (Fujifilm Wako Pure Chemical, Osaka, Japan), diluted in 0.1 vol% formic acid in distilled water (Kanto Chemical, Tokyo, Japan). Mobile phase B was 0.1% formic acid in acetonitrile (Kanto Chemical, Tokyo, Japan). The elution profile was as follows: 0–7.5 min, 75%–60% B; 7.5–8.5 min, 60%–20% B; 8.5–11.5 min, 20% B; followed by washing with 0% B for 2 min and equilibration with 75% B for 4.8 min. The column oven temperature was set at 35 °C. The flow rate was 350 μL/min (26). Several HMOs were identified by comparing their retention times and mass-to-charge ratios (*m/z*) to commercial standards: 2′-fucosyllactose (2′FL; Biosynth, Staad, Switzerland), 3-fucosyllactose (3FL; Biosynth), 3′-sialyllactose (3′SL; Biosynth), 6′-sialyllactose (6′SL; Biosynth), lactodifucotetraose (LDFT; IsoSep, Tullinge, Sweden), lacto-N-tetraose (LNT; Biosynth), lacto-N-neotetraose (LNnT; Biosynth), lacto-N-fucopentaose-I (LNFPI; Biosynth), lacto-N-fucopentaose-II (LNFPII; Biosynth), lacto-N-fucopentaose-III (LNFPIII; IsoSep), sialyllacto-N-tertaose a (LSTa; IsoSep), sialyllacto-N-tertaose b (LSTb; IsoSep), sialyllacto-N-tertaose c (LSTc; IsoSep), lacto-N-difucohexaose-I (LNDFHI; Dextra Laboratories, Collegiate Square, UK), difucosyllacto-N-hexaose b (DFLNHb; IsoSep), and disialyllacto-N-tetraose (DSLNT; Dextra Laboratories). The details of the detection conditions for LC-MS are provided in the Supplementary Tables S1, S3. Quantification was performed using a calibration curve (2′FL; 75–9,600 mg/L, 3FL; 37.5–4,800 mg/L, 3′SL; 37.5–4,800 mg/L, 6′SL; 37.5–4,800 mg/L, LDFT; 37.5–4,800 mg/L, LNT; 37.5–9,600 mg/L, LNnT; 37.5–4,800 mg/L, LNFPI; 37.5–4,800 mg/L, LNFPII; 75–4,800 mg/L, LNFPIII; 37.5–4,800 mg/L, LSTa; 37.5–4,800 mg/L, LSTb; 37.5–4,800 mg/L, LSTc; 37.5–4,800 mg/L, LNDFHI; 150–4,800 mg/L, DFLNHb; 75–4,800 mg/L, DSLNT; 37.5–4,800 mg/L). In this study, individuals with 2′FL concentrations in breast milk of less than 75 mg/L and an LNFPI concentration of less than 37.5 mg/L were classified as non-secretors.

### Purification and analysis of human-breast-milk-derived EVs

The method of collecting MEVs from human breast milk was adapted from a previous study (23). A 1:100 volume of acetic acid (Kanto Chemical) was added to defatted milk, which was then centrifuged at 4,500 × *g* for 30 min at 4 °C to remove residual fat and casein. The whey was passed through a 0.22-μm filter to remove remaining cell debris. A qEV column (qEV original/35; Izon Science, Christchurch, New Zealand) was used according to the manufacturer’s protocols. Briefly, after washing the column with phosphate-buffered saline (PBS), 0.5 mL of whey was applied to the top of the qEV column, and 0.5-mL fractions were collected. In cases where less than 0.5 mL of whey could be recovered, the entire volume of collected whey was used for EV extraction out of necessity. Five fractions rich in MEVs (fractions 7–11) were pooled, and the MEVs were collected. An example of changes in fraction concentration is shown in Supplementary Figure S2. MEVs were visualized using transmission electron microscopy (TEM) at the Hanaichi Ultra Structure Research Institute (Okazaki, Japan). For nanoparticle tracking analysis, MEVs were assessed using a NanoSight LM10B-HSF device (Malvern Panalytical, Malvern, UK) equipped with a 488-nm excitation laser. Measurements were recorded at camera level 14 with a detection threshold of 5.

### Analysis of RNAs

RNA was extracted from MEVs and purified using a miRNeasy Serum/Plasma Kit (Qiagen, Hilden, Germany), as described previously (27). RNA quantity and integrity were assessed using an RNA 6000 Pico Kit (Agilent Technologies, Santa Clara, CA) with an Agilent 2100 Bioanalyzer (Agilent Technologies). Extracted RNA was generated and labeled with cyanine-3 using a miRNA Complete Labeling Reagent and Hyb Kit (Agilent Technologies) and hybridized to a SurePrint G3 Human miRNA 8 × 60 K (Agilent Technologies) according to the manufacturer’s instructions. Each sample was measured using standardized volumes of breast milk. Fluorescence signals were detected using the Agilent SureScan Microarray Scanner Extraction software version 12.0.3.1 (Agilent Technologies) according to the manufacturer’s instructions. Raw data from the feature extraction software were exported to the GeneSpring GX version 14.9 (Agilent Technologies). To identify differentially expressed genes in the microarray and reduce noise, each fluorescence signal dataset was normalized using a median shift algorithm (shifted to the 75th percentile) with background correction to the median of all samples. Only genes with normalized signals detected on all microarrays were considered present. All microarray data were deposited in the National Center for Biotechnology Information Gene Expression Omnibus (GEO) and are accessible through GEO Series accession number GSE294523.

### Extraction of HMOs from EVs

EVs extracted using the qEV column (a mix of fractions 7–11) were combined, and the amount extracted from whey equivalent to 60 μL was concentrated using a SpeedVac (45 °C, 3 h). The samples were then purified by acetonitrile precipitation and filtered under the conditions described in the “Analysis of HMOs” section.

### Statistical analysis

JMP software (version Pro 14.0.0, SAS Institute, Cary, NC) was used for statistical analysis. Since the study is ongoing and not all participants have completed all time points, the sample sizes differed across time points. All factorial analyses were conducted using the rankFD package (version 0.1.1) in R (version 4.5.1). Non-parametric Wald-type statistics were calculated to assess the main effects and interactions. Spearman’s rank correlation coefficients were calculated to assess the associations between variables using R. For comparisons between two groups, Student *t*-tests were conducted. To account for multiple testing, *p*-values were adjusted using the Benjamini–Hochberg procedure for false discovery rate (FDR) control, with FDR set at 5% (28).

## Results

### Characteristics of the participants

The characteristics of the participants are listed in Table 1. Overall, mothers were 31.9 ± 2.6 years old at the time of data collection and had a pre-pregnancy body mass index (BMI) of 20.4 ± 1.5. Among the 16 participants, 11 were classified as secretors (Table 1). The protein concentration in breast milk decreased over time during lactation, but the change was not statistically significant. Fat concentrations decreased significantly but peaked at 2 months postpartum. Carbohydrates, total solids, and energy did not change over time during the course of lactation (Supplementary Figure S1).

**Table 1:** Characteristics of the participants.

Maternal background	Recorded data
Participants (*n*)	16
Primipara (*n*)	9
Age (years)	31.9 ± 2.6
Height (cm)	157.2 ± 3.5
Pre-pregnancy body weight (kg)	50.3 ± 3.6
Pre-pregnancy BMI^1^	20.4 ± 1.5
Gestation period (weeks)	38.9 ± 0.9
Body weight at delivery (kg)	58.7 ± 0.9
Weight gain (kg)	8.4 ± 4.1
Average total fat in breast milk (g/100 mL)	3.3 ± 1.7
Average true protein in breast milk (g/100 mL)	1.0 ± 0.2
Average carbohydrate in breast milk (g/100 mL)	8.1 ± 0.2
Average total solids in breast milk (g/100 mL)	12.9 ± 1.8
Average energy in breast milk (g/100 mL)	68.7 ± 16.1
Secretor, non-secretor (*n*, *n*)^2^	11, 5

One of the 16 participants did not complete the questionnaire. ^1^BMI, body mass index. ^2^Secretor type was defined by the presence or absence of 2′-fucosyllactose or lacto-N-fucopentaose I.

### HMO concentrations during lactation

**Figure 1:**
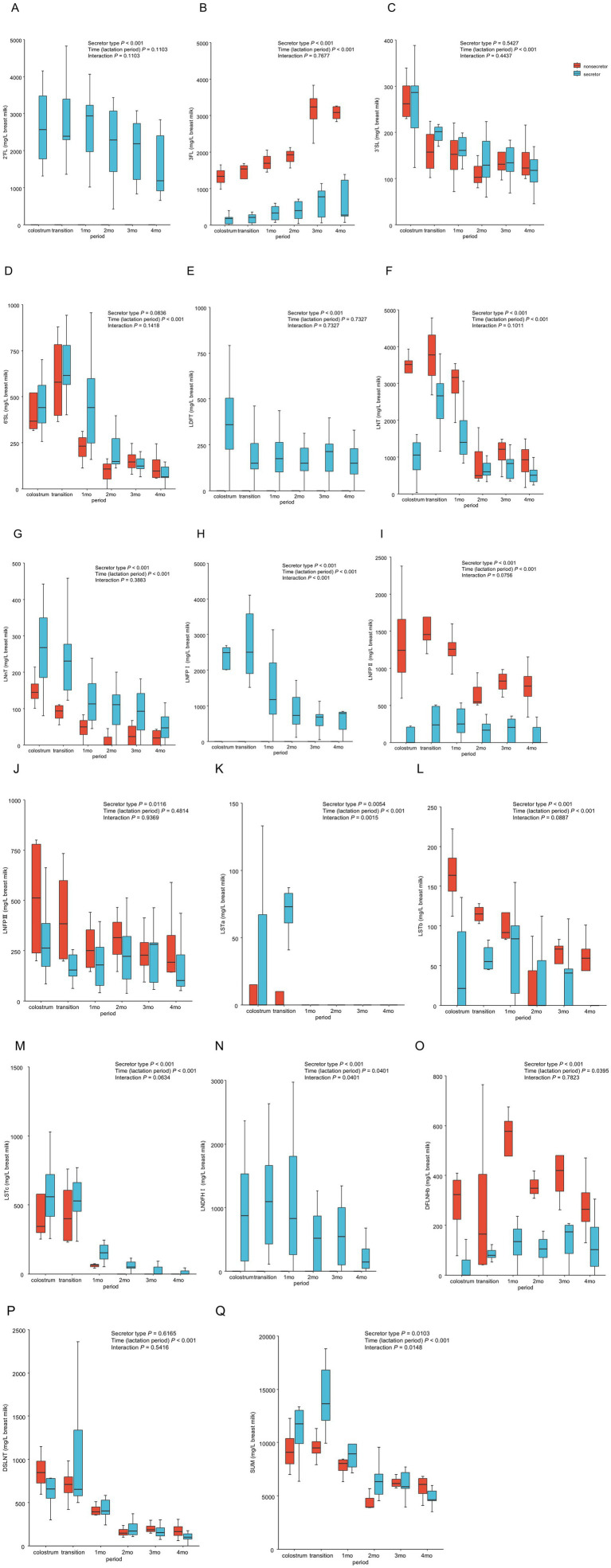
Concentrations of HMOs from colostrum to 4 months postpartum in breast milk. **(A)** 2′FL; **(B)** 3FL; **(C)** 3′SL; **(D)** 6′SL; **(E)** LDFT; **(F)** LNT; **(G)** LNnT; **(H)** LNFPI; **(I)** LNFPII; **(J)** LNFPIII; **(K)** LSTa; **(L)** LSTb; **(M)** LSTc; **(N)** LNDFHI; **(O)** DFLNHb; **(P)** DSLNT; and **(Q)** sum of 16 HMOs. Non-parametric Wald-type statistics were calculated to assess the main effects and interactions. *n* = 12 (colostrum); *n* = 12 (transition); *n* = 14 (1 month); *n* = 14 (2 months); *n* = 13 (3 months); *n* = 11 (over 4 months). HMOs, human milk oligosaccharides; 2′FL, 2-fucosyllactose; 3FL, 3-fucosyllactose; 3′SL, 3′-sialyllactose; 6′SL, 6′-sialyllactose; LDFT, lactodifucotetraose; LNT, lacto-N-tetraose; LNnT, lacto-N-neotetraose; LNFPI-III, lacto-N-fucopentaose I-III; LSTa-c, sialyl-lacto-N-tetraose a-c; Lacto-N-difucohexaose-I, LNDFHI; DFLNHb, difucosyl-lacto-N-hexaose b; and DSLNT, disialyllacto-N-tetraose.

**Figure 2:**
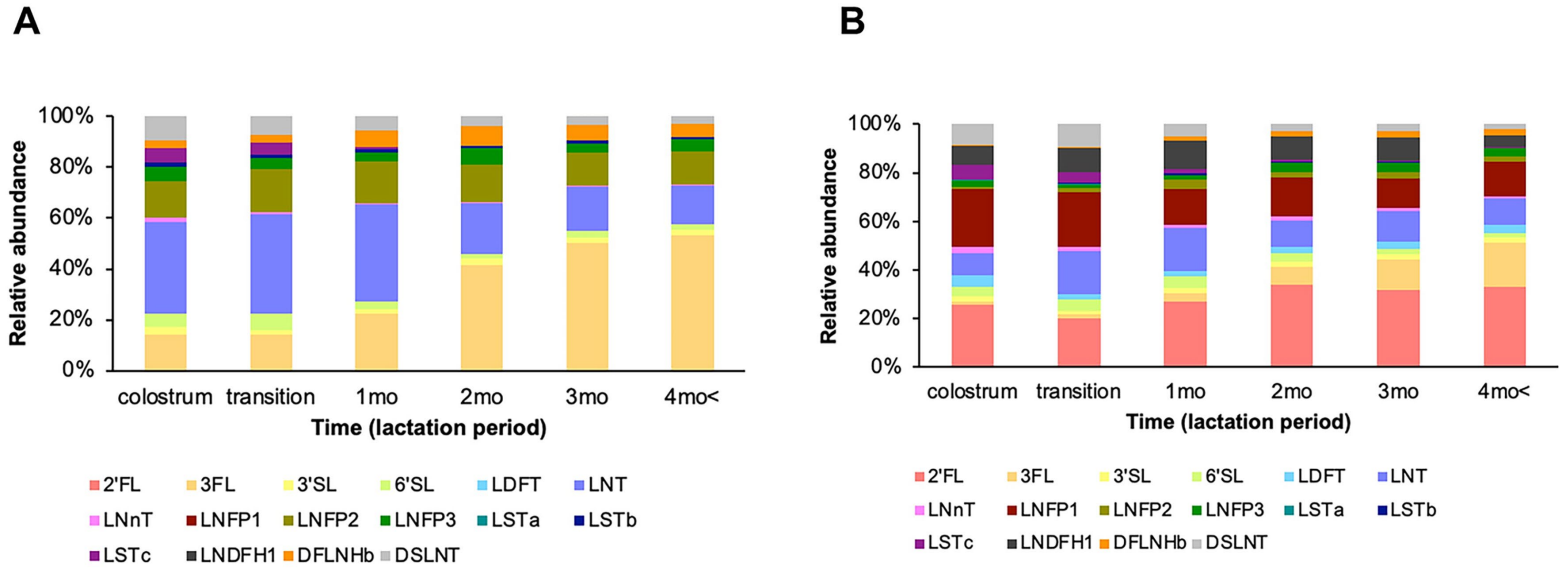
Relative abundances of HMOs from colostrum to 4 months postpartum in breast milk. The order of HMOs in the legends represented the order of colors in the figure. **(A)** Non-secretors and **(B)** secretors. *n =* 12 (colostrum); *n =* 12 (transition); *n =* 14 (1 month); *n =* 14 (2 months); *n =* 13 (3 months); *n =* 11 (over 4 months). Abbreviations: HMOs, human milk oligosaccharides; 2′FL, 2-fucosyllactose; 3FL, 3-fucosyllactose; 3′SL, 3′-sialyllactose; 6′SL, 6′-sialyllactose; LDFT, lactodifucotetraose; LNT, lacto-N-tetraose; LNnT, lacto-N-neotetraose; LNFPI-III, lacto-N-fucopentaose I-III; LSTa-c, sialyl-lacto-N-tetraose a-c; Lacto-N-difucohexaose-I, LNDFHI; DFLNHb, difucosyl-lacto-N-hexaose b; and DSLNT, disialyllacto-N-tetraose.

For 13 HMOs (3FL, 3′SL, 6′SL, LNT, LNnT, LNFPI, LNFPII, LSTa, LSTb, LSTc, LNDFHI, DFLNHb, and DSLNT) and the total sum of HMOs, there was a significant main effect of time on concentration (Figures 1B-D, F-I, K-Q). The concentrations of these HMOs significantly decreased from colostrum to 4 months postpartum, with the exception of 3FL (Figure 1B). For 2′FL, 3FL, LDFT, LNT, LNnT, LNFPI, LNFPII, LNFPIII, LSTa, LSTb, LSTc, LNDFHI, DFLNHb, and the total sum of HMOs, there was a significant main effect of secretor type on concentration (Figures 1A, B, E–O, Q). Among these HMOs, the concentrations of 2’FL, LDFT, LNnT, LNFPI, LSTa, LSTc, LNDFHI, and the total sum of HMOs were higher in secretors than in non-secretors (Figures 1A, E, G, H, K, M, N, Q), and those of 3FL, LNT, LNFPII, LNFPIII, LSTb, and DFLNHb were higher in non-secretors than in secretors (Figures 1B, F, I, J, L, O). For LNFPI, LSTa, LNDFHI, and total HMO, there were significant interaction effects of time and secretor type on concentrations (Figures 1H, K, N, Q). The LNFPI concentration decreased by 76% from colostrum to 4 months in secretors (Figure 1H). The LSTa concentration decreased by 81% from colostrum to 1 month in secretors (Figure 1K). The LNDFHI concentration decreased by 76% from colostrum to 4 months in secretors but did not change in non-secretors (Figure 1N). The total HMO concentration decreased by 59% from colostrum to 4 months in secretors but remained unchanged in non-secretors (Figure 1Q). In non-secretors, the proportion of LNT was highest during early lactation, and that of 3FL was highest during late lactation (Figure 2A). In secretors, the proportions of 2′FL and LNFPI were highest during early lactation. During late lactation, the proportion of 2′FL was highest, followed by 3FL and LNFPI (Figure 2B).

### Characteristics of EVs in breast milk during lactation

**Figure 3:**
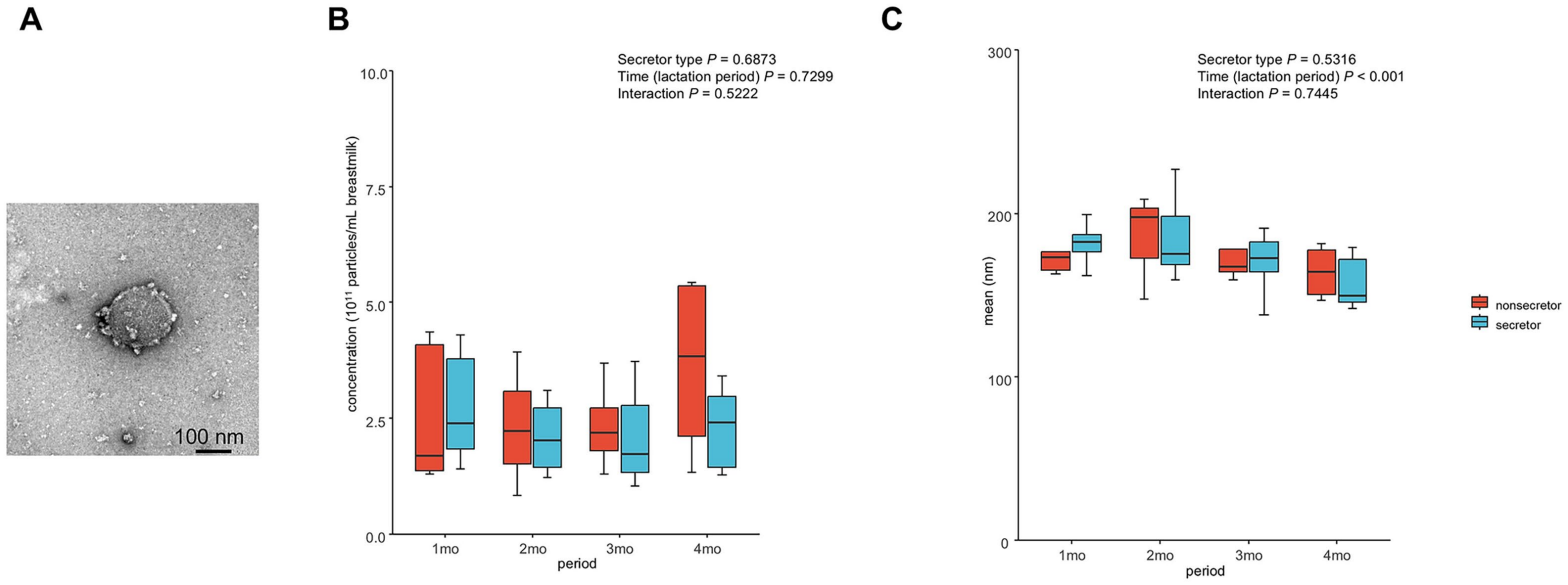
Characteristics of extracellular vesicles in breast milk. **(A)** Transmission electron micrographs of MEVs. **(B)** Numbers of MEVs during lactation as determined by nanoparticle tracking analysis. **(C)** Size of MEVs during lactation by nanoparticle tracking analysis. Non-parametric Wald-type statistics were calculated to assess the main effects and interactions. *n =* 12 (colostrum); *n =* 12 (transition); *n =* 15 (1 month); *n =* 14 (2 months); *n =* 12 (3 months); *n =* 11 (over 4 months). Abbreviation: MEVs, milk-derived extracellular vesicles.

The morphology of MEVs in breast milk was visualized by TEM (Figure 3A). Regarding the number of MEVs, there were no significant main effects of time and secretor type (Figure 3B). Regarding the mean size of MEVs, there was a significant main effect of time (Figure 3C).

### miRNAs in breast milk during lactation

**Figure 4:**
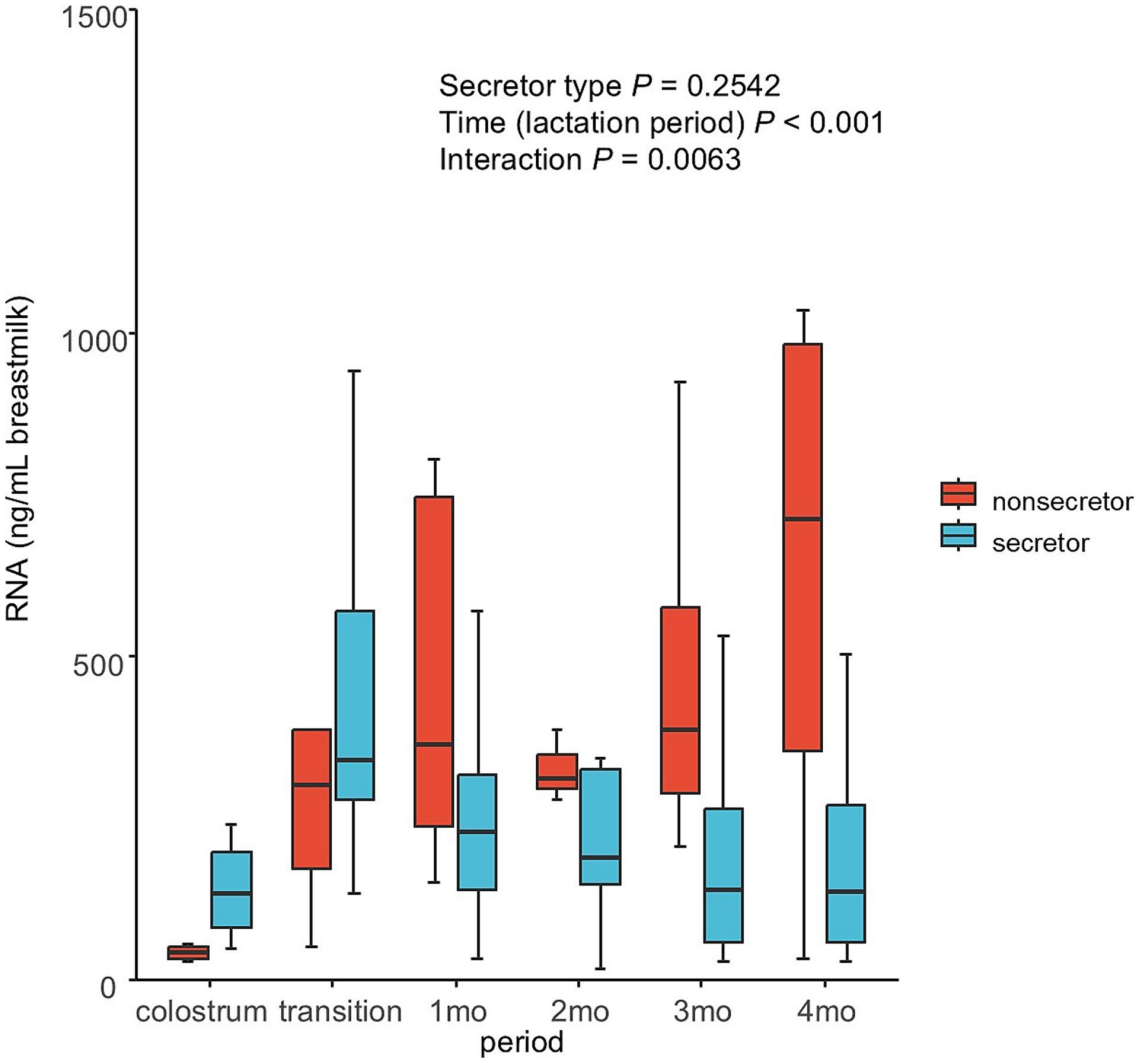
RNA concentrations in human breast MEVs. RNA concentration in breast MEVs during lactation as analyzed using the Agilent 2100 Bioanalyzer. Non-parametric Wald-type statistics were calculated to assess the main effects and interactions. *n =* 12 (colostrum); *n =* 12 (transition); *n =* 15 (1 month); *n =* 14 (2 months); *n =* 12 (3 months); *n =* 11 (over 4 months).

**Figure 5:**
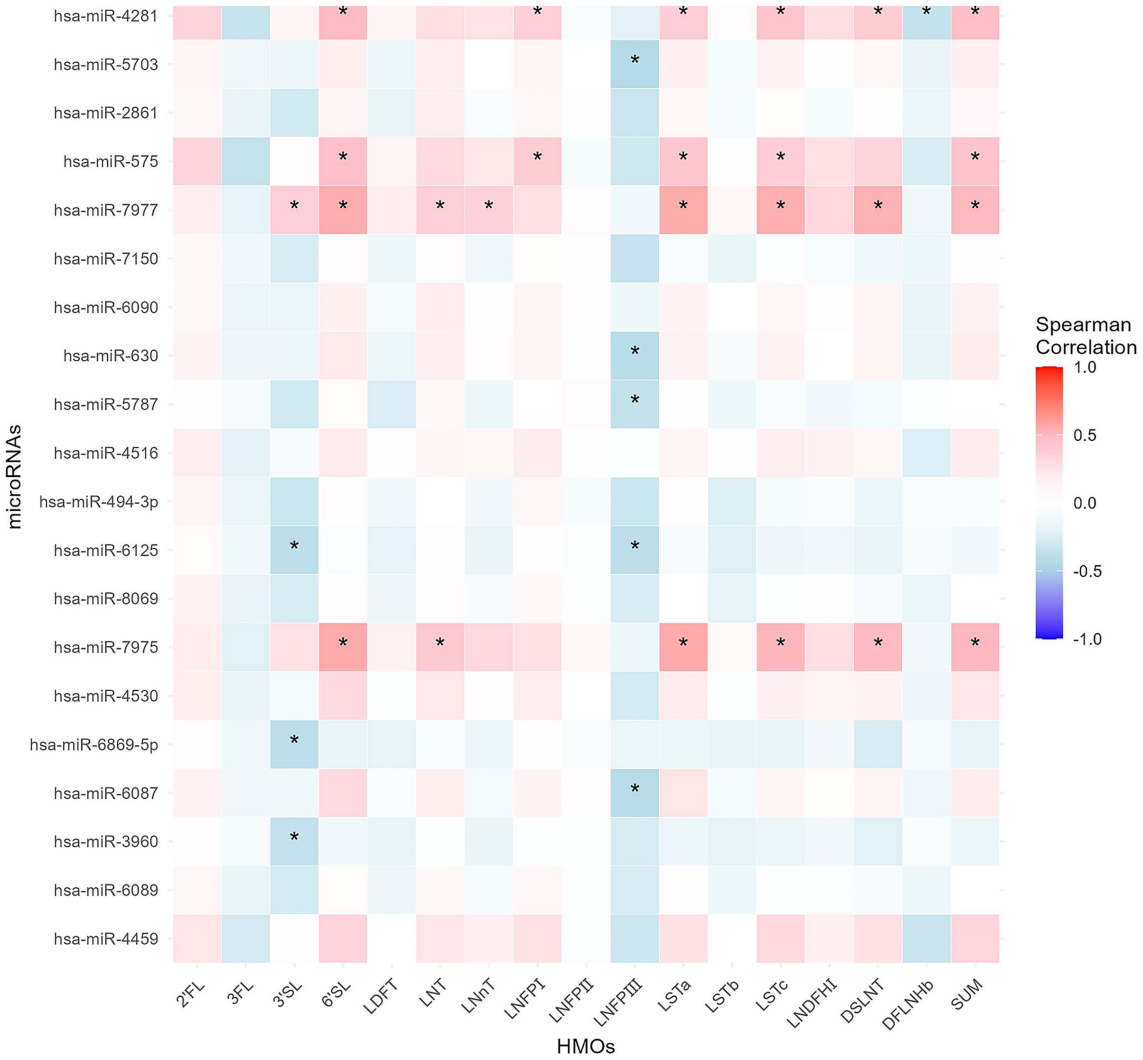
A heatmap showing Spearman correlations among HMO concentrations and the top 20 highly expressed microRNAs. Red shading indicates positive Spearman correlations and blue shading indicates negative Spearman correlations, calculated with the Benjamini–Hochberg method; color saturation represents the strength of the correlation. Stars indicate statistical significance (*p < 0.05).

**Figure 6:**
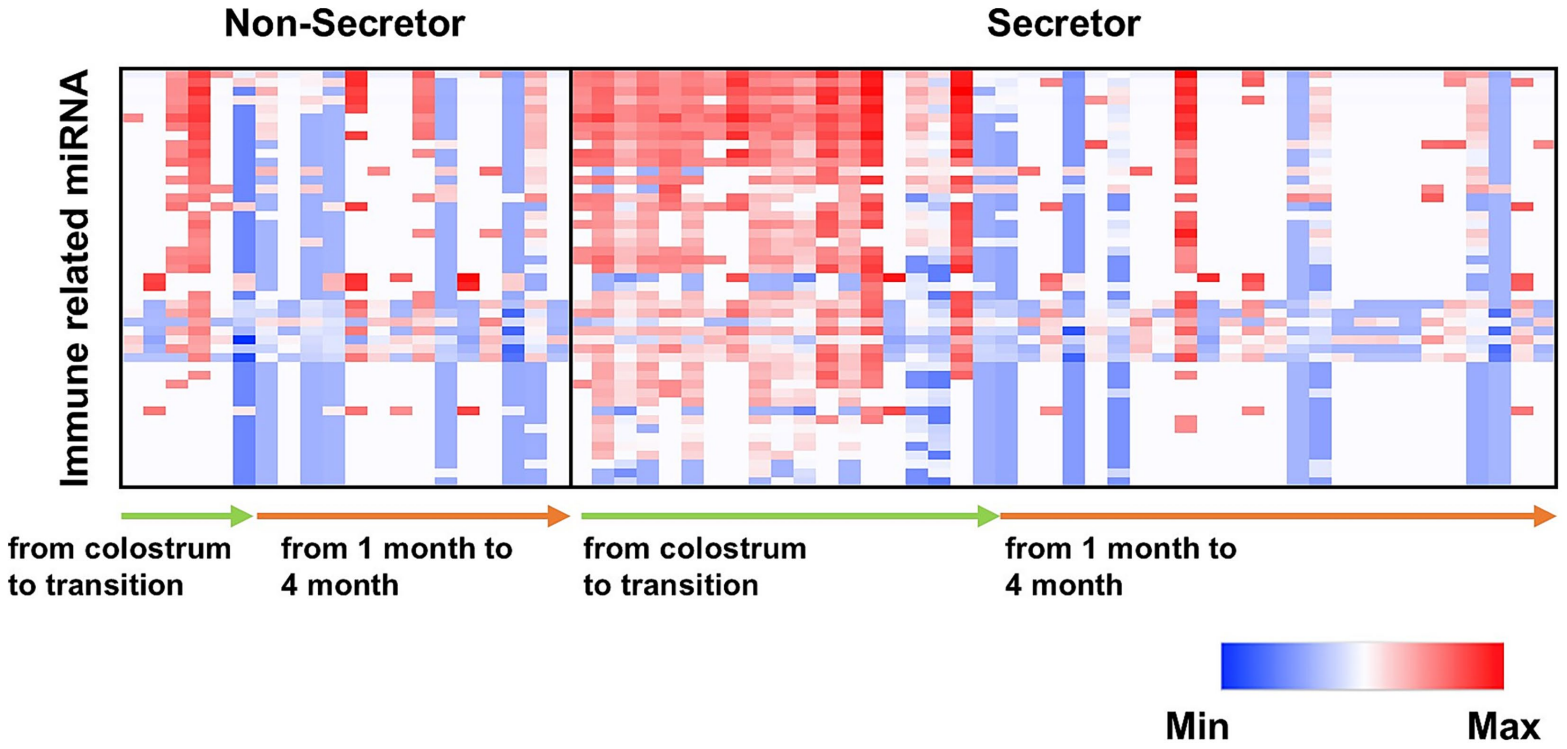
Relationships between immunity- and development-related miRNAs detected in breast MEVs and secretor types. Heatmap of the normalized array data for immunity- and development-related miRNAs detected in secretors and non-secretors.

For RNA concentration in breast milk, there was a significant main effect of time and a significant interaction between time and secretor type (Figure 4). The miRNA microarray analysis identified 1,232 miRNAs in MEVs; the top 20 (based on raw signal) are listed in Table 2. Moreover, the correlations between the top 20 most highly expressed miRNAs and 16 types of HMOs in breast milk were analyzed (Figure 5). The results revealed that LNFPIII exhibited negative correlations with 5 miRNAs in the top 20 miRNAs (hsa-miR-5703, hsa-miR-630, hsa-miR-5787, hsa-miR-6125, and hsa-miR-6087). 3′SL exhibited negative correlations with three types of miRNAs (hsa-miR-6125, hsa-miR-6869-5p, and hsa-miR-3960). Notably, among the top 20 miRNAs, hsa-miR-4281, hsa-miR-575, hsa-miR-7977, and hsa-miR-7975 showed either positive or negative correlations with more than five different HMOs. The top 20 normalized miRNA array data, to evaluate expression levels independent of individual differences in RNA content, are listed in Supplementary Table S2. Among the detected miRNAs, those reported to be related to development or immunity were selected (Supplementary Table S3) (19, 29–32). The expression levels of these miRNAs tended to be higher in secretors during the colostrum-to-transition milk period (Figure 6).

**Table 2:** Top 20 most highly expressed miRNAs in MEVs.

Rank	Name
1	hsa-miR-4459
2	hsa-miR-6089
3	hsa-miR-3960
4	hsa-miR-6087
5	hsa-miR-6869-5p
6	hsa-miR-4530
7	hsa-miR-7975
8	hsa-miR-8069
9	hsa-miR-6125
10	hsa-miR-494-3p
11	hsa-miR-4516
12	hsa-miR-5787
13	hsa-miR-630
14	hsa-miR-6090
15	hsa-miR-7150
16	hsa-miR-7977
17	hsa-miR-575
18	hsa-miR-2861
19	hsa-miR-5703
20	hsa-miR-4281

### HMOs in EVs

**Figure 7:**
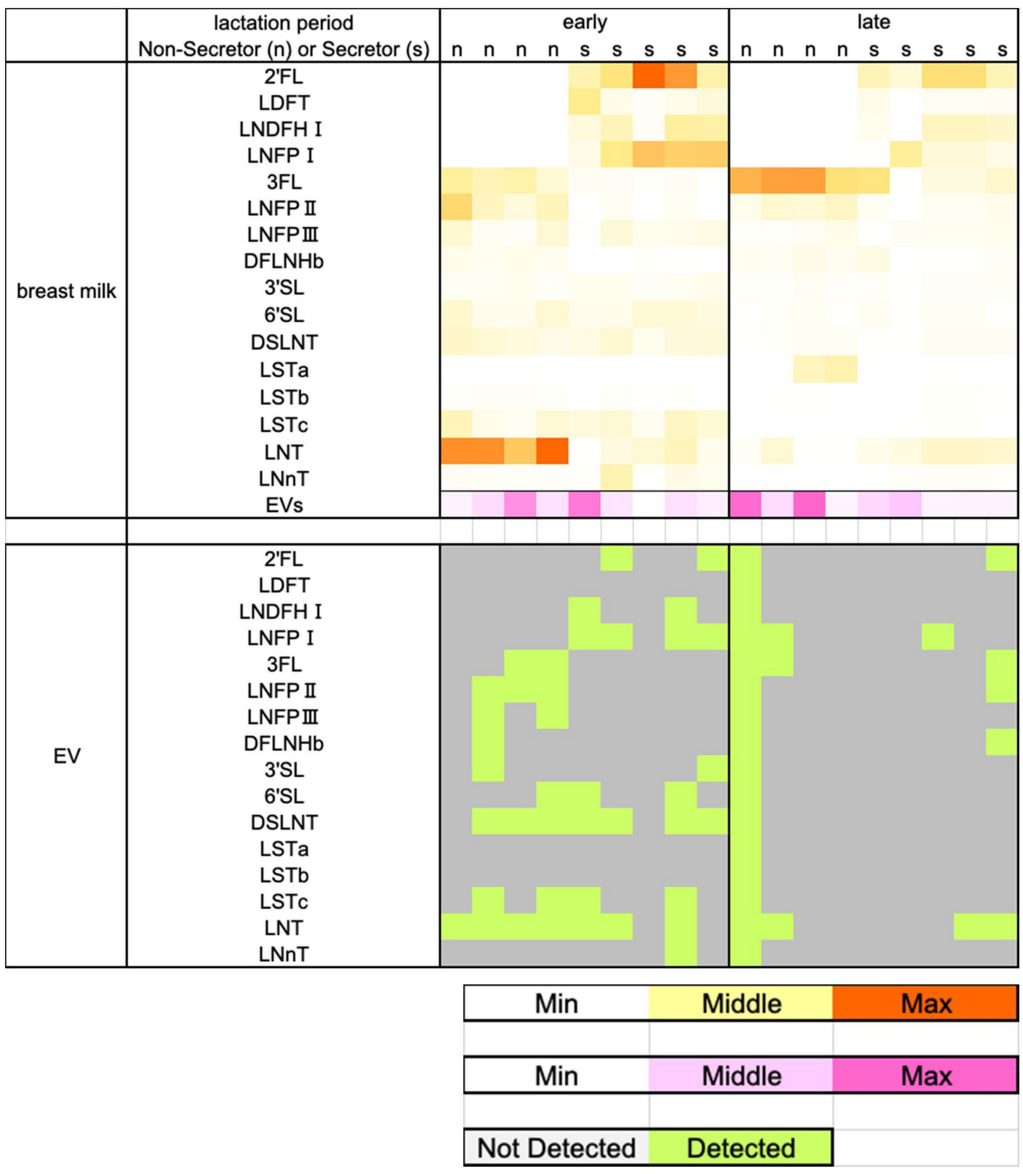
HMOs are detected in breast milk or extracellular vesicles. 2′FL, 3FL, 3′SL, 6′SL, LDFT, LNT, LNnT, LNFPI, LNFPII, LNFPIII, LSTa, LSTb, LSTc, LNDFHI, DFLNHb, and DSLNT in breast milk or extracellular vesicles were detected by tandem mass spectrometry. Abbreviations: 2′FL, 2-fucosyllactose; 3FL, 3-fucosyllactose; 3′SL, 3′-sialyllactose; 6′SL, 6′-sialyllactose; LDFT, lactodifucotetraose; LNT, lacto-N-tetraose; LNnT, lacto-N-neotetraose; LNFPI-III, lacto-N-fucopentaose I-III; LSTa-c, sialyl-lacto-N-tetraose a-c; Lacto-N-difucohexaose-I, LNDFHI; DFLNHb, difucosyl-lacto-N-hexaose b; and DSLNT, disialyllacto-N-tetraose.

HMOs were detected in at least one of the same MEVs during the colostrum-to-transition milk period (early lactation) and at 3–4 months postpartum (late lactation) (Figure 7). Several HMOs were detected in MEVs more frequently during early than late lactation. Eleven HMOs (2′FL, LNDFHI, LNFPI, LNFPII, LNFPIII, 3′SL, 6′SL, DSLNT, LSTc, LNT, and LNnT) were detected at least as frequently or more frequently during early lactation. During early lactation, α1–2-fucosylated HMOs, with the exception of LDFT (2′FL, LNDFHI, and LNFPI), were detected in MEVs only from secretors. In contrast, α1–4-fucosylated HMOs and α1–6-fucosylated HMOs (3FL, LNFPII, LNFPIII, and DFLNHb) were detected only in MEVs from non-secretors. However, these trends disappeared during late lactation.

## Discussion

**Figure 8:**
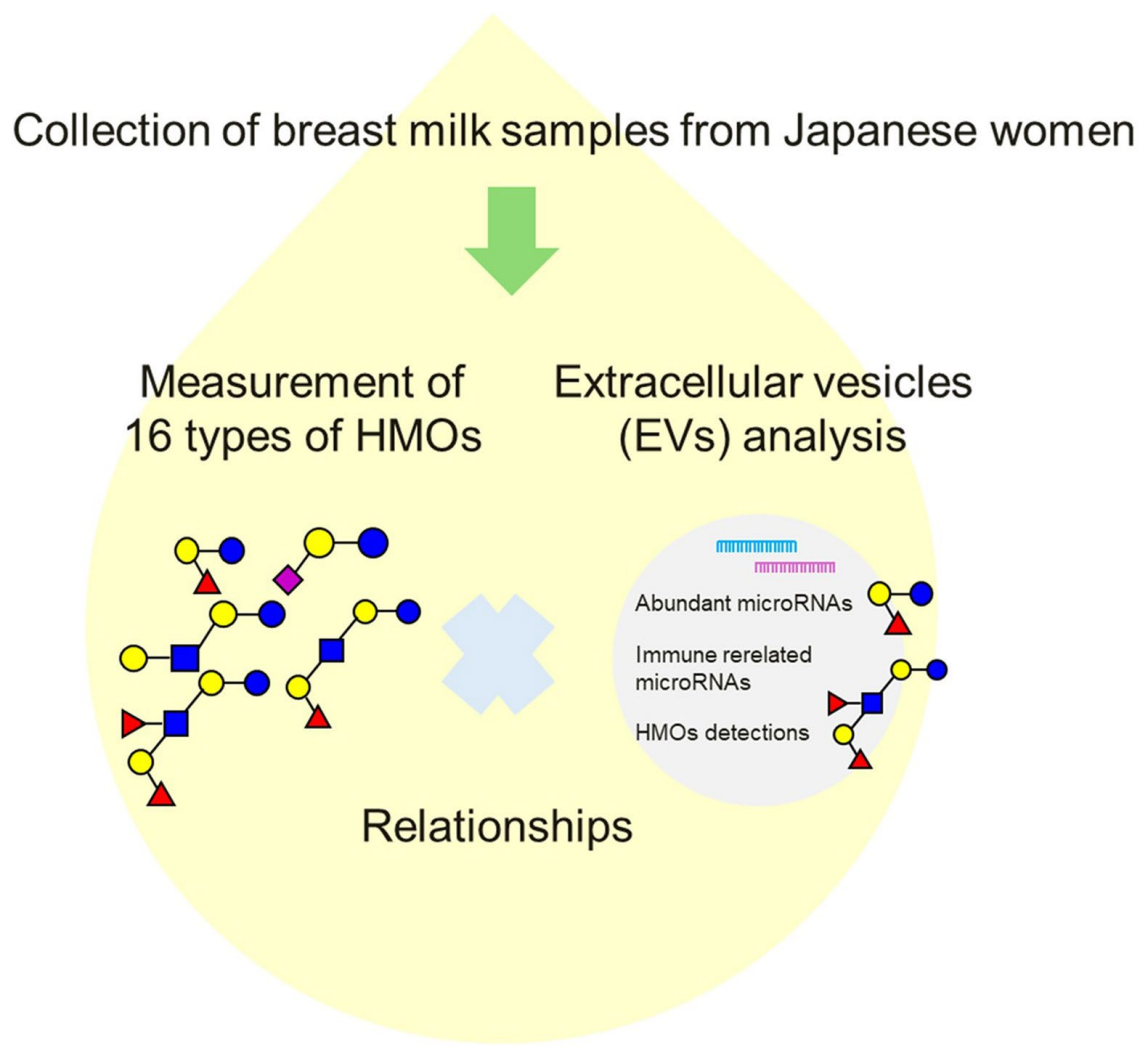
The overview of this study.

We investigated the longitudinal changes in HMOs and MEVs in lactating Japanese women and analyzed the relationships between HMOs and miRNAs in MEVs. The overall scheme of this experiment is shown in Figure 8.

In this study, the relative abundance of 3FL increased over the course of lactation in both non-secretors and secretors, which was consistent with the findings of a previous report (16). The absolute concentrations of HMOs have been reported not to increase, with the exception of 3FL (12, 15, 33, 34). Some studies have reported an increase in 3′SL in addition to 3FL (13, 16). In this study, only 3FL increased over time. It is possible that factors such as ethnicity or geographic differences contribute to the differences among studies (11). Our findings suggest that the absolute concentrations of most HMOs decrease over the course of lactation; the exceptions are 3FL (which increased) and 2′FL, LDFT, and LNFPIII (which did not change significantly). HMO concentrations also differ by ethnicity and geographic differences. The proportion of secretors is significantly higher in Peru than in rural Gambia, Washington (United States), Ghana, and rural Ethiopia. This finding suggests that HMO concentrations vary according to geographic, ethnic, genetic, and environmental factors. Asia is highly ethnically diverse, making it important to evaluate the concentrations of HMOs in breast milk among Asian populations (11). The average absolute concentrations of 16 HMOs in breast milk have been compared among populations in China, Malaysia, Singapore, and Indonesia (14, 33, 35–37). The concentrations of 2′FL, 3FL, 6′SL, and LNT in Japanese women in this study were similar to those in other Asian populations. The concentration of 3′SL was higher during early lactation in Japanese, Indonesian, and Singaporean women but tended to be lower in Malaysian women. The concentration of LNnT in breast milk was higher in Malaysian women compared with other Asian women (including the Japanese women in this study). The LSTc concentrations varied geographically during the colostrum-to-transition milk period, but they were similar after the first month. We have no data on the average absolute concentrations of the other HMOs (LDFT, LNFPI, LNFPII, LNFPIII, LSTa, LSTb, LNDFHI, DFLNHb, and DSLNT) in other Asian populations, making it difficult to compare them. When developing infant formula, it may be necessary to consider regional and ethnic variations in HMO concentration. The decreases in the absolute concentrations of most HMOs (3′SL, 6′SL, LNT, LNnT, LNFPI, LNFPII, LSTa, LSTb, LSTc, LNDFHI, DFLNHb, and DSLNT) during lactation suggest that they have important biological functions in the early months of life. Notably, 3′SL and 6′SL are positively associated with brain development (38). LNT and LNFPI inhibit the growth of *Streptococcus agalactiae*, which causes invasive bacterial infections in newborns (39). The production of butyrate, a short-chain fatty acid resulting from the fermentation of LNnT and 2′FL (40), is implicated in the regulation of the growth and function of innate and adaptive immune cells. Calicivirus diarrhea was less frequent in infants whose mothers’ milk contained high levels of LNDFHI (41). DSLNT protects against necrotizing enterocolitis (42). The biological functions of LNFPII, LSTa, LSTb, LSTc, and DFLNHb remain unknown because their limited availability precludes human intervention studies and cell culture experiments. Because LNFPI, LNFPII, and LNFPIII are structural isomers, LNFPII and LNFPIII may exhibit biological activities similar to those of LNFPI. Being sialylated, LSTa, LSTb, and LSTc may be essential for brain and cognitive development. In addition, sialylated milk oligosaccharides may act as prebiotics, stimulating the growth of gut bacteria. The structure of DFLNHb is complex, and its biological functions remain unclear. The fact that the absolute concentrations of 3FL, 2′FL, LDFT, and LNFPIII were stable or increased during lactation suggests that they have important biological functions beyond the first few months of life. For example, 3FL can prevent norovirus infection (43). 3FL and LDFT attenuate tumor necrosis factor-*α*-induced IL-8 secretion by immature epithelial cells (44). 2′FL stimulates brain development, improves cognitive outcomes (45, 46), and promotes a rapid increase in infant body weight (47). Further research is needed to analyze the functions of these HMOs.

The mean number of MEVs was 2.7 × 10^11^/mL throughout lactation, as reported previously (48–50). The mean size of MEVs was 184.4 nm throughout lactation, as indicated in prior studies (50, 51). In this study, MEV size decreased significantly over time, but significant changes were not observed in secretor type. The changes in MEV size throughout lactation suggest that the composition and characteristics of MEVs changed during lactation. Colostrum and mature milk exert various protective effects on inflammation (52), consistent with the morphological changes reported in this study. The mean concentration of RNA in MEVs was 358.5 ng/mL in breast milk throughout lactation, which is 1.5-fold higher than previously reported (19). These differences are likely a result of the use of different lactation periods and MEV purification processes. It has been reported that colostrum contains more RNA than mature milk (27, 53). In this study, we extracted MEVs, unlike previous studies. Furthermore, prior studies focused on other species, such as rats and cows. A previous study that involved the extraction of RNA from human MEVs showed no significant difference in RNA concentration between colostrum and mature milk (54). Consequently, species differences and the presence or absence of the MEV extraction processes may influence the results.

A microarray analysis identified 1,232 miRNAs in MEVs, the functions of many of which were unknown. Among the top 20 miRNAs, 12 (hsa-miR-4459, hsa-miR-6089, hsa-miR-3960, hsa-miR-6869-5p, hsa-miR-4530, hsa-miR-7975, hsa-miR-8069, hsa-miR-6125, hsa-miR-6090, hsa-miR-7150, hsa-miR-5703, and hsa-miR-4281) have not been functionally characterized. The remaining miRNAs (hsa-miR-6087, hsa-miR-494-3p, hsa-miR-4516, hsa-miR-5787, hsa-miR-630, hsa-miR-7977, hsa-miR-575, and hsa-miR-2861) have a variety of functions. For example, hsa-miR-6087 significantly downregulates endoglin, a marker of endothelial cells (55). Additionally, hsa-miR-494-3p attenuates the transcription of *HtrA3*, thereby increasing the inflammatory response in hypoxia/reoxygenation-treated HK2 cells (56). Hsa-miR-4516 enhances the cisplatin resistance of ovarian cancer cells by suppressing GAS7 (57). Hsa-miR-5787 prevents the proliferation and migration of macrophages and attenuates LPS/TLR4-mediated inflammations via NF-κB (58). Hsa-miR-630 regulates the production of underglycosylated IgA1 in the tonsils by targeting Toll-like receptor 4 (TLR4) in IgA nephropathy (59). Hsa-miR-7977 functions as part of the pathways involved in diabetic wound repair (60). Hsa-miR-575 modulates cell cycle progression and proliferation (61). Finally, hsa-miR-2861 functions as a tumor suppressor by targeting the EGFR/AKT2/CCND1 pathway in human papillomavirus type 16 E6-induced cervical cancer (62). miRNAs are present in various proportions in MEVs. miRNAs can regulate each other’s expression, leading to a highly complex mechanism of action. A correlation analysis between the top 20 raw signal-based miRNAs and 16 types of HMOs revealed that 3′SL and LNFPIII exhibited negative correlations with a large number of miRNAs. miRNAs related to immunity or development (19, 29–32, 63) were abundant among the top 20 normalized miRNA array data (Supplementary Table S2). Moreover, immune- or development-related miRNAs were more prevalent in the colostrum of secretors. To our knowledge, no previous study has examined the association between secretor type and MEVs. Secretor status may influence infant immunity by affecting the gut microbiota (64). Our data implicate the miRNAs in MEVs in development and immunity in secretors and non-secretors. This is the first study to report that the secretor type may influence the miRNA composition of MEVs. HMOs were detected in MEVs from at least one participant in this study. MEVs are heterogeneous in composition and have various cellular origins. The biological functions of HMOs detected in MEVs are unclear. In this study, although HMOs were successfully detected in EVs, the amount was extremely small compared to the total amount present in breast milk. Further investigation is needed to determine whether the HMOs detected are being transported by EVs or are co-eluted from the free HMOs in milk. On the other hand, as shown in Figure 7, even when both the HMO concentration and EV concentration in breast milk are high, HMOs are not necessarily detected in EVs. Therefore, rather than co-elution, we expect that HMOs may be incorporated within EVs or bound to the EV membrane as glycans.

This study was limited by the small number of participants. Compared to previous studies, the narrower window during the lactation period enabled a more detailed quantitative analysis. Additionally, the concentrations of HMOs in MEVs were considerably lower than in breast milk, which limited the assessment of the concentrations of HMOs in MEVs. Therefore, devices and methods for detecting HMOs with higher sensitivity are needed. Additionally, breast milk samples were collected at each participant’s home, and consequently, there was a period during which the samples were stored at −20 °C prior to further analysis. Therefore, it is not possible to completely rule out the possibility that storage conditions may have influenced the state of RNA.

In conclusion, this is the first report describing longitudinal changes in HMOs and MEVs in Japanese women. The findings suggest a relationship between secretor type and immune-related miRNAs in MEVs. Moreover, the relationship between HMOs and miRNAs in MEVs suggests that miRNAs in MEVs are linked to the functions of HMOs. Further research is needed to determine the functions of HMOs and miRNAs in MEVs in infants.

## Data Availability

The datasets presented in this study can be found in online repositories. The names of the repository/repositories and accession number(s) can be found at: https://www.ncbi.nlm.nih.gov/geo/, GSE294523.

## References

[1] Victora CG Bahl R Barros AJD França GVA Horton S Krasevec J . Breastfeeding in the 21st century: epidemiology, mechanisms, and lifelong effect. Lancet. (2016) 387:475–90. doi: 10.1016/S0140-6736(15)01024-7 26869575

[2] Oddy WH. Breast feeding and respiratory morbidity in infancy: a birth cohort study. Arch Dis Child. (2003) 88:224–8. doi: 10.1136/adc.88.3.224, 12598384 PMC1719488

[3] Tarrant RC Younger KM Sheridan-Pereira M White MJ Kearney JM. The prevalence and determinants of breast-feeding initiation and duration in a sample of women in Ireland. Public Health Nutr. (2010) 13:760–70. doi: 10.1017/S1368980009991522, 19758484

[4] Eidelman AI Schanler RJ. Breastfeeding and the use of human milk. Pediatrics. (2012) 129:e827–41. doi: 10.1542/peds.2011-3552, 22371471

[5] Morrison DJ Preston T. Formation of short chain fatty acids by the gut microbiota and their impact on human metabolism. Gut Microbes. (2016) 7:189–200. doi: 10.1080/19490976.2015.1134082, 26963409 PMC4939913

[6] Zhao Q Elson CO. Adaptive immune education by gut microbiota antigens. Immunology. (2018) 154:28–37. doi: 10.1111/imm.12896, 29338074 PMC5904715

[7] Inchingolo F Inchingolo AM Latini G Ferrante L de Ruvo E Campanelli M . Difference in the intestinal microbiota between breastfeed infants and infants fed with artificial milk: a systematic review. Pathogens. (2024) 13:533. doi: 10.3390/pathogens13070533, 39057760 PMC11280328

[8] Bode L. Human milk oligosaccharides: every baby needs a sugar mama. Glycobiology. (2012) 22:1147–62. doi: 10.1093/glycob/cws074, 22513036 PMC3406618

[9] Dinleyici M Barbieur J Dinleyici EC Vandenplas Y. Functional effects of human Milk oligosaccharides (HMOs). Gut Microbes. (2023) 15:2186115. doi: 10.1080/19490976.2023.2186115, 36929926 PMC10026937

[10] Chaturvedi P Warren CD Altaye M Morrow AL Ruiz-Palacios G Pickering LK . Fucosylated human Milk oligosaccharides vary between individuals and over the course of lactation. Glycobiology. (2001) 11:365–72. doi: 10.1093/glycob/11.5.365, 11425797

[11] McGuire MK Meehan CL McGuire MA Williams JE Foster J Sellen DW . What’s Normal? Oligosaccharide concentrations and profiles in Milk produced by healthy women vary geographically. Am J Clin Nutr. (2017) 105:1086–100. doi: 10.3945/ajcn.116.139980, 28356278 PMC5402033

[12] Coppa G Pierani P Zampini L Carloni I Carlucci A Gabrielli O. Oligosaccharides in human milk during different phases of lactation. Acta Paediatr. (1999) 88:89–94. doi: 10.1111/j.1651-2227.1999.tb01307.x, 10569230

[13] Ferreira AL Alves R Figueiredo A Alves-Santos N Freitas-Costa N Batalha M . Human Milk oligosaccharide profile variation throughout postpartum in healthy women in a Brazilian cohort. Nutrients. (2020) 12:790. doi: 10.3390/nu12030790, 32192176 PMC7146368

[14] Sprenger N Lee LY De Castro CA Steenhout P Thakkar SK. Longitudinal change of selected human Milk oligosaccharides and association to infants’ growth, an observatory, single Center, longitudinal cohort study. PLoS One. (2017) 12:e0171814. doi: 10.1371/journal.pone.0171814, 28182762 PMC5300226

[15] Thurl S Munzert M Henker J Boehm G Mller-Werner B Jelinek J . Variation of human Milk oligosaccharides in relation to Milk groups and lactational periods. Br J Nutr. (2010) 104:1261–71. doi: 10.1017/S0007114510002072, 20522272

[16] Plows JF Berger PK Jones RB Alderete TL Yonemitsu C Najera JA . Longitudinal changes in human Milk oligosaccharides (HMOs) over the course of 24 months of lactation. J Nutr. (2021) 151:876–82. doi: 10.1093/jn/nxaa427, 33693851 PMC8030713

[17] Sato K Nakamura Y Fujiyama K Ohneda K Nobukuni T Ogishima S . Absolute quantification of eight human Milk oligosaccharides in breast Milk to evaluate their concentration profiles and associations with infants’ neurodevelopmental outcomes. J Food Sci. (2024) 89:10152–70. doi: 10.1111/1750-3841.17597, 39656795 PMC11673463

[18] van Leeuwen SS. Challenges and pitfalls in human milk oligosaccharide analysis. Nutrients. (2019) 11:2684. doi: 10.3390/nu11112684, 31698698 PMC6893418

[19] Kosaka N Izumi H Sekine K Ochiya T. MicroRNA as a new immune-regulatory agent in breast Milk. Silence. (2010) 1:7. doi: 10.1186/1758-907X-1-7, 20226005 PMC2847997

[20] Li X Su L Zhang X Chen Q Wang Y Shen Z . Recent advances on the function and purification of milk exosomes: a review. Front Nutr. (2022) 9:871346. doi: 10.3389/fnut.2022.871346, 35757254 PMC9219579

[21] He S Liu G Zhu X. Human breast Milk-derived exosomes may help maintain intestinal epithelial barrier integrity. Pediatr Res. (2021) 90:366–72. doi: 10.1038/s41390-021-01449-y, 33731816

[22] Näslund TI Paquin-Proulx D Paredes PT Vallhov H Sandberg JK Gabrielsson S. Exosomes from breast Milk inhibit HIV-1 infection of dendritic cells and subsequent viral transfer to CD4+ T cells. AIDS. (2014) 28:171–80. doi: 10.1097/QAD.0000000000000159, 24413309

[23] Morozumi M Izumi H Shimizu T Takeda Y. Comparison of isolation methods using commercially available kits for obtaining extracellular vesicles from cow Milk. J Dairy Sci. (2021) 104:6463–71. doi: 10.3168/jds.2020-19849, 33714584

[24] He Y He Z Leone S Liu S. Milk exosomes transfer oligosaccharides into macrophages to modulate immunity and attenuate adherent-invasive e. coli (Aiec) infection. Nutrients. (2021) 13:3198. doi: 10.3390/nu13093198, 34579075 PMC8472098

[25] Holzhausen EA Patterson WB Wong BH Kim S Kupsco A Howe CG . Associations between human Milk EV-MiRNAs and oligosaccharide concentrations in human Milk. Front Immunol. (2024) 15:1463463. doi: 10.3389/fimmu.2024.1463463, 39635519 PMC11614774

[26] Zhang W Wang T Chen X Pang X Zhang S Obaroakpo JU . Absolute quantification of twelve oligosaccharides in human Milk using a targeted mass spectrometry-based approach. Carbohydr Polym. (2019) 219:328–33. doi: 10.1016/j.carbpol.2019.04.092, 31151532

[27] Izumi H Kosaka N Shimizu T Sekine K Ochiya T Takase M. Time-dependent expression profiles of MicroRNAs and MRNAs in rat Milk whey. PLoS One. (2014) 9:e88843. doi: 10.1371/journal.pone.0088843, 24533154 PMC3923055

[28] Benjamini Y Hochberg Y. Controlling the false discovery rate: a practical and powerful approach to multiple testing. J R Stat Soc Series B Stat Methodol. (1995) 57:289–300. doi: 10.1111/j.2517-6161.1995.tb02031.x

[29] Perri M Lucente M Cannataro R De Luca IF Gallelli L Moro G . Variation in immune-related MicroRNAs profile in human Milk amongst lactating women. MicroRNA. (2018) 7:107–14. doi: 10.2174/2211536607666180206150503, 29412128

[30] Zhou Q Li M Wang X Li Q Wang T Zhu Q . Immune-related MicroRNAs are abundant in breast Milk exosomes. Int J Biol Sci. (2012) 8:118–23. doi: 10.7150/ijbs.8.118, 22211110 PMC3248653

[31] Tingö L Ahlberg E Johansson L Pedersen SA Chawla K Sætrom P . Non-coding RNAs in human breast milk: a systematic review. Front Immunol. (2021) 12:725323. doi: 10.3389/fimmu.2021.725323, 34539664 PMC8440964

[32] Na RS E GX Sun W Sun XW Qiu XY Chen LP . Expressional analysis of immune-related MiRNAs in breast Milk. Genet Mol Res. (2015) 14:11371–6. doi: 10.4238/2015.September.25.4, 26436378

[33] Wu J Wu S Huo J Ruan H Xu X Hao Z . Systematic characterization and longitudinal study reveal distinguishing features of human milk oligosaccharides in China. Curr Dev Nutr. (2020) 4:nzaa113. doi: 10.1093/cdn/nzaa113, 32734137 PMC7382630

[34] Samuel TM Binia A de Castro CA Thakkar SK Billeaud C Agosti M . Impact of maternal characteristics on human milk oligosaccharide composition over the first 4 months of lactation in a cohort of healthy European mothers. Sci Rep. (2019) 9:11767. doi: 10.1038/s41598-019-48337-4, 31409852 PMC6692355

[35] Ma L McJarrow P Jan Mohamed HJB Liu X Welman A Fong BY. Lactational changes in the human milk oligosaccharide concentration in Chinese and Malaysian mothers’ milk. Int Dairy J. (2018) 87:1–10. doi: 10.1016/j.idairyj.2018.07.015

[36] Sudarma V Sunardi D Marzuki NS Munasir Z Asmarinah Hidayat A . Human Milk oligosaccharide profiles and the secretor and Lewis gene status of Indonesian lactating mothers. Pediatr Gastroenterol Hepatol Nutr. (2023) 26:266–76. doi: 10.5223/pghn.2023.26.5.266, 37736221 PMC10509021

[37] Ning Y Xun Y Fong B McJarrow P Ma L Jan Mohamed HJ . Analysis of twelve human Milk oligosaccharides over fifteen months post-partum in human Milk from Chinese mothers. Heliyon. (2024) 10:e39293. doi: 10.1016/j.heliyon.2024.e39293, 39640655 PMC11620220

[38] Cho S Zhu Z Li T Baluyot K Howell BR Hazlett HC . Human Milk 3’-Sialyllactose is positively associated with language development during infancy. Am J Clin Nutr. (2021) 114:588–97. doi: 10.1093/ajcn/nqab103, 34020453 PMC8326052

[39] Lin AE Autran CA Szyszka A Escajadillo T Huang M Godula K . Human Milk oligosaccharides inhibit growth of group B Streptococcus. J Biol Chem. (2017) 292:11243–9. doi: 10.1074/jbc.M117.789974, 28416607 PMC5500792

[40] Šuligoj T Vigsnæs LK Van den Abbeele P Apostolou A Karalis K Savva GM . Effects of human Milk oligosaccharides on the adult gut microbiota and barrier function. Nutrients. (2020) 12:1–21. doi: 10.3390/nu12092808, 32933181 PMC7551690

[41] Morrow AL Ruiz-Palacios GM Altaye M Jiang X Lourdes Guerrero M Meinzen-Derr JK . Human Milk oligosaccharides are associated with protection against Diarrhea in breast-fed infants. J Pediatr. (2004) 145:297–303. doi: 10.1016/j.jpeds.2004.04.054, 15343178

[42] Jantscher-Krenn E Zherebtsov M Nissan C Goth K Guner YS Naidu N . The human Milk oligosaccharide Disialyllacto-N-Tetraose prevents necrotising enterocolitis in neonatal rats. Gut. (2012) 61:1417–25. doi: 10.1136/gutjnl-2011-301404, 22138535 PMC3909680

[43] Weichert S Koromyslova A Singh BK Hansman S Jennewein S Schroten H . Structural basis for norovirus inhibition by human Milk oligosaccharides. J Virol. (2016) 90:4843–8. doi: 10.1128/jvi.03223-15, 26889023 PMC4836343

[44] Cheng L Kong C Wang W Groeneveld A Nauta A Groves MR . The human milk oligosaccharides 3-FL, lacto-N-neotetraose, and LDFT attenuate tumor necrosis factor-α induced inflammation in fetal intestinal epithelial cells in vitro through shedding or interacting with tumor necrosis factor receptor 1. Mol Nutr Food Res. (2021) 65:e2000425. doi: 10.1002/mnfr.202000425, 33465830 PMC8047892

[45] Oliveros E Ramirez M Vazquez E Barranco A Gruart A Delgado-Garcia JM . Oral supplementation of 2′-Fucosyllactose during lactation improves memory and learning in rats. J Nutr Biochem. (2016) 31:20–7. doi: 10.1016/j.jnutbio.2015.12.014, 27133420

[46] Berger PK Plows JF Jones RB Alderete TL Yonemitsu C Poulsen M . Human Milk oligosaccharide 2’-Fucosyllactose links feedings at 1 month to cognitive development at 24 months in infants of Normal and overweight mothers. PLoS One. (2020) 15:e0228323. doi: 10.1371/journal.pone.0228323, 32049968 PMC7015316

[47] Larsson MW Lind MV Laursen RP Yonemitsu C Larnkjær A Mølgaard C . Human Milk oligosaccharide composition is associated with excessive weight gain during exclusive breastfeeding—an explorative study. Front Pediatr. (2019) 7:297. doi: 10.3389/fped.2019.00297, 31380329 PMC6657391

[48] Vaswani K Mitchell MD Holland OJ Qin Koh Y Hill RJ Harb T . A method for the isolation of exosomes from human and bovine Milk. J Nutr Metab. (2019) 2019:1–6. doi: 10.1155/2019/5764740, 31885909 PMC6914892

[49] Gómez-Ferrer M Amaro-Prellezo E Albiach-Delgado A Ten-Domenech I Kuligowski J Sepúlveda P. Identification of omega-3 oxylipins in human milk-derived extracellular vesicles with pro-resolutive actions in gastrointestinal inflammation. Front Immunol. (2023) 14:1293737. doi: 10.3389/fimmu.2023.1293737, 38054009 PMC10694275

[50] Kupsco A Prada D Valvi D Hu L Petersen MS Coull B . Human Milk extracellular vesicle MiRNA expression and associations with maternal characteristics in a population-based cohort from the Faroe Islands. Sci Rep. (2021) 11:5840. doi: 10.1038/s41598-021-84809-2, 33712635 PMC7970999

[51] Bickmore DC Miklavcic JJ. Characterization of extracellular vesicles isolated from human Milk using a precipitation-based method. Front Nutr. (2020) 7:22. doi: 10.3389/fnut.2020.00022, 32232046 PMC7082312

[52] Gao R Zhang R Qian T Peng X He W Zheng S . A comparison of exosomes derived from different periods breast Milk on protecting against intestinal organoid injury. Pediatr Surg Int. (2019) 35:1363–8. doi: 10.1007/s00383-019-04562-6, 31576466

[53] Izumi H Kosaka N Shimizu T Sekine K Ochiya T Takase M. Bovine Milk contains MicroRNA and messenger RNA that are stable under degradative conditions. J Dairy Sci. (2012) 95:4831–41. doi: 10.3168/jds.2012-5489, 22916887

[54] Freiría-Martínez L Iglesias-Martínez-Almeida M Rodríguez-Jamardo C Rivera-Baltanás T Comís-Tuche M Rodrígues-Amorím D . Human breast Milk MicroRNAs, potential players in the regulation of nervous system. Nutrients. (2023) 15:3284. doi: 10.3390/nu15143284, 37513702 PMC10384760

[55] Yoo JK Kim J Choi S Noh HM Kwon YD Yoo H . Discovery and characterization of novel MicroRNAs during endothelial differentiation of human embryonic stem cells. Stem Cells Dev. (2012) 21:2049–57. doi: 10.1089/scd.2011.0500, 22142236 PMC3396149

[56] Gong Q Shen Z Sheng Z Jiang S Ge S. Hsa-MiR-494-3p attenuates gene HtrA3 transcription to increase inflammatory response in hypoxia/reoxygenation HK2 cells. Sci Rep. (2021) 11:1665. doi: 10.1038/s41598-021-81113-x, 33462352 PMC7814133

[57] Pan X Shi X Zhang H Chen Y Zhou J Shen F . Exosomal MiR-4516 derived from ovarian Cancer stem cells enhanced cisplatin tolerance in ovarian Cancer by inhibiting GAS7. Gene. (2024) 927:148738. doi: 10.1016/j.gene.2024.148738, 38955306

[58] Bao Z Zhang S Li X. MiR-5787 attenuates macrophages-mediated inflammation by targeting TLR4/NF-ΚB in ischemic cerebral infarction. NeuroMolecular Med. (2021) 23:363–70. doi: 10.1007/s12017-020-08628-w, 33165670

[59] Liu C Ye M-Y Yan W-Z Peng X-F He L-Y Peng Y-M. MicroRNA-630 regulates Underglycosylated IgA1 production in the tonsils by targeting TLR4 in IgA nephropathy. Front Immunol. (2020) 11:563699. doi: 10.3389/fimmu.2020.563699, 33324395 PMC7725902

[60] Ji Z Wang J Yang S Tao S Shen C Wei H . Graphene oxide accelerates diabetic wound repair by inhibiting apoptosis of ad-MSCs via Linc00324/miR-7977/STK4 pathway. FASEB J. (2022) 36:e22623. doi: 10.1096/fj.202201079RR, 36269304

[61] Wang Q Lu W Lu L Wu R Wu D. MiR-575/RIPK4 Axis modulates cell cycle progression and proliferation by inactivating the Wnt/β-catenin Signaling pathway through inhibiting RUNX1 in Colon Cancer. Mol Cell Biochem. (2024) 479:1747–66. doi: 10.1007/s11010-024-04938-w, 38480605

[62] Xu J Wan X Chen X Fang Y Cheng X Xie X . MiR-2861 acts as a tumor suppressor via targeting EGFR/AKT2/CCND1 pathway in cervical Cancer induced by human papillomavirus virus 16 E6. Sci Rep. (2016) 6:28968. doi: 10.1038/srep28968, 27364926 PMC4929448

[63] Ahlberg E Martí M Govindaraj D Severin E Duchén K Jenmalm MC . Immune-related MicroRNAs in breast Milk and their relation to regulatory T cells in breastfed children. Pediatr Allergy Immunol. (2023) 34:e13952. doi: 10.1111/pai.13952, 37102392

[64] Gurung M Schlegel BT Rajasundaram D Fox R Bode L Yao T . Microbiota from human infants consuming secretors or non-secretors mothers’ milk impacts the gut and immune system in mice. mSystems. (2024) 9:e0029424. doi: 10.1128/msystems.00294-24, 38530054 PMC11019842

