# Supplementary material for: Changes in and relationships between human milk oligosaccharides and microRNAs in milk-derived extracellular vesicles during the first 4 months of lactation
Source: Front Nutr. 2025 Dec 22;12:1694093. doi: 10.3389/fnut.2025.1694093 (PMC12766977; doi:10.3389/fnut.2025.1694093)
Supplement: Supplementary file 1 [file Data_Sheet_1.docx]

**Supplemental Information**

**(A)**


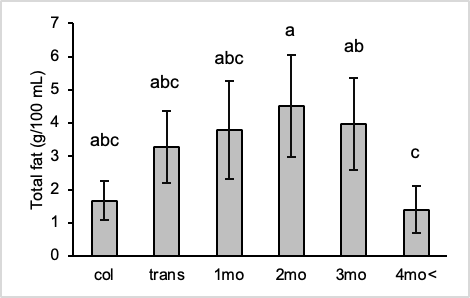


**(B)**


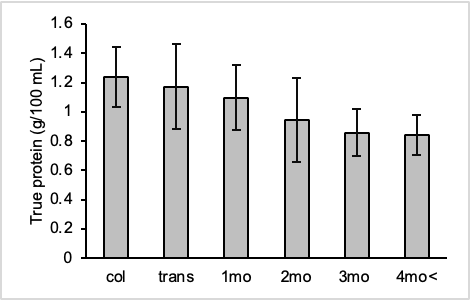


**a**

**a**

**a**

**a**

**a**

**a**

**(C)**


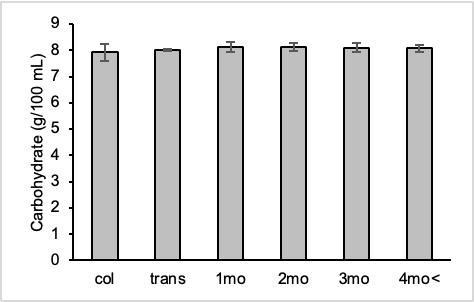


**a**

**a**

**a**

**a**

**a**

**a**

**(D)**


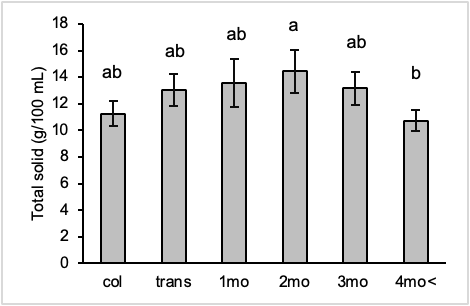


**(E)**


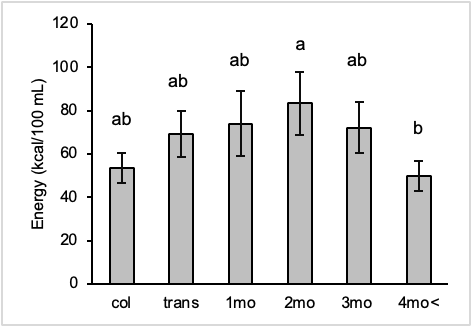


Supplemental Figure 1. Measurement of actual macronutrient content of the milk. (A) Fat; (B) protein; (C) carbohydrate; (D) solid; and (E) energy in breast milk during lactation were analyzed by Human Milk Analyzer. Data are expressed as means ± SD (n = 3-10), which were analyzed by one-way ANOVA followed by a Tukey–Kramer HSD test. Values with different letters are significantly different.

Supplemental Figure 2. Particle numbers in each fraction of qEV. The typical behavior of particle numbers in the collected fraction were shown. Particle numbers were determined by measuring the collected mEVs using a NanoSight LM10B-HSF (Malvern Panalytical, Great Malvern, UK) equipped with a 488-nm excitation laser. Measurements were recorded at camera level 14 and a detection threshold of 5.


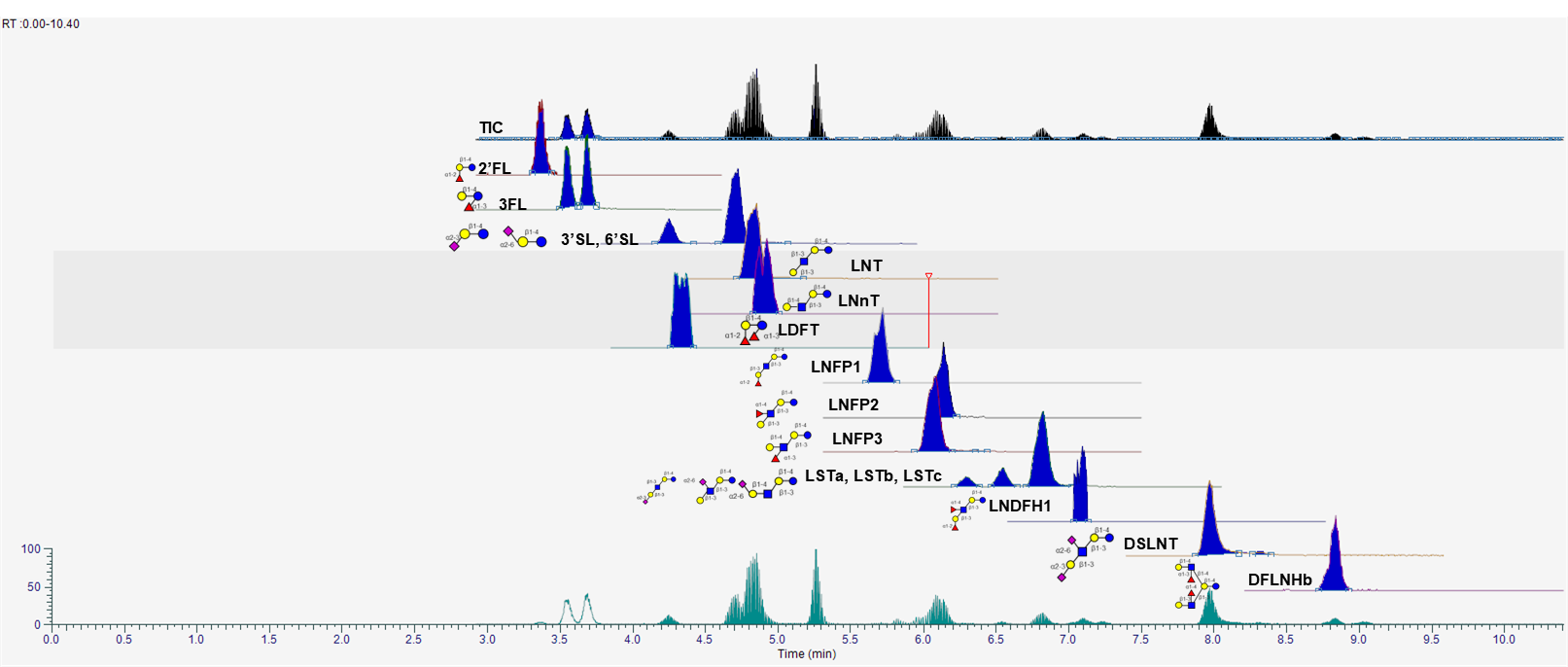


Supplemental Figure 3. The selected ion chromatograms with labelled glycans analyzed in PRM mode
